# (*E*)-2-Meth­oxy-6-(thia­zol-2-ylimino­meth­yl)phenol

**DOI:** 10.1107/S1600536809040410

**Published:** 2009-10-10

**Authors:** Wenkuan Li, Handong Yin, Liyuan Wen, Weidong Fan, Jing Li

**Affiliations:** a College of Chemistry and Chemical Engineering, Liaocheng University, Shandong 252059, People’s Republic of China

## Abstract

The title compound, C_11_H_10_N_2_O_2_S, displays an *E* configuration about the C=N bond. The mean planes of the thia­zole and benzene rings make a dihedral angle of 9.32 (18)°. Intra­molecular O—H⋯N hydrogen bonds are found in the crystal structure.

## Related literature

For general background to Schiff bases, see: Lv *et al.* (2006 ▶); Tarafder *et al.* (2002 ▶); Zhou *et al.* (2009 ▶).
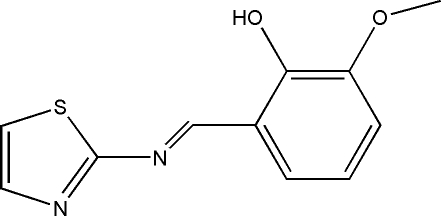


## Experimental

### 

#### Crystal data

C_11_H_10_N_2_O_2_S
*M* *_r_* = 234.27
Monoclinic, 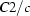

*a* = 24.765 (3) Å
*b* = 4.9619 (8) Å
*c* = 20.238 (2) Å
β = 117.931 (2)°
*V* = 2197.2 (5) Å^3^
*Z* = 8
Mo *K*α radiation
μ = 0.28 mm^−1^
*T* = 298 K
0.29 × 0.18 × 0.17 mm

#### Data collection

Siemens SMART CCD area-detector diffractometer
Absorption correction: multi-scan (*SADABS*; Sheldrick, 1996 ▶) *T*_min_ = 0.923, *T*_max_ = 0.954
5338 measured reflections
1920 independent reflections
1139 reflections with *I* > 2σ(*I*)
*R*_int_ = 0.034

#### Refinement

*R*[*F* ^2^ > 2σ(*F* ^2^)] = 0.044
*wR*(*F* ^2^) = 0.110
*S* = 1.02
1920 reflections
146 parameters
H-atom parameters constrained
Δρ_max_ = 0.19 e Å^−3^
Δρ_min_ = −0.23 e Å^−3^

### 

Data collection: *SMART* (Siemens, 1996 ▶); cell refinement: *SAINT* (Siemens, 1996 ▶); data reduction: *SAINT*; program(s) used to solve structure: *SHELXS97* (Sheldrick, 2008 ▶); program(s) used to refine structure: *SHELXL97* (Sheldrick, 2008 ▶); molecular graphics: *SHELXTL* (Sheldrick, 2008 ▶); software used to prepare material for publication: *SHELXTL*.

## Supplementary Material

Crystal structure: contains datablocks I, global. DOI: 10.1107/S1600536809040410/jj2011sup1.cif

Structure factors: contains datablocks I. DOI: 10.1107/S1600536809040410/jj2011Isup2.hkl

Additional supplementary materials:  crystallographic information; 3D view; checkCIF report

## Figures and Tables

**Table 1:** Hydrogen-bond geometry (Å, °)

*D*—H⋯*A*	*D*—H	H⋯*A*	*D*⋯*A*	*D*—H⋯*A*
O1—H1⋯N2	0.82	1.91	2.627 (3)	146

**Fig. 1.**
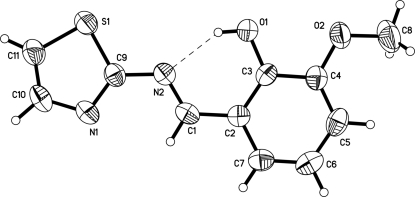
The molecular structure of the title molecule, with 50% probability displacement ellipsoids. An O—H..N intramolecular hydrogen bond is shown as a dashed line.

**Fig. 2.**
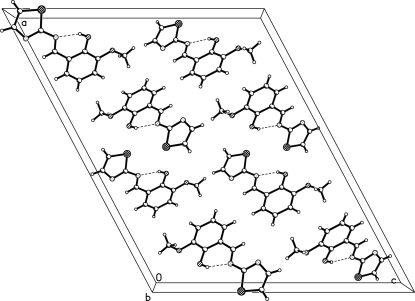
The molecular packing of the title compound, viewed along the b axis. Intramolecular hydrogen bonds are shown as dashed lines.

## supplementary crystallographic information

### Comment

Schiff bases have been extensively researched because of their important applications in coordination chemistry, catalysis and biological processes. (Lv *et al.* 2006, Tarafder *et al.* 2002, Zhou *et al.* 2009). Owing to the importance of these Schiff base analogue compounds, we report here the crystal structure of the title compound, C_11_H_10_N_2_O_2_S_1_, (I).

The title compoud  was prepared by the condensation reaction of 2-hydroxy-3-methoxybenzaldehyde with an equimolar quantity of thiazol-2 -amine (Fig. 1). The structure of (I) shows a *trans* or E configuration about the C=N bond. The dihedral angle between the mean planes of the thiazole and benzene rings is 9.32 (18) °. Intramolecular O1—H1···N2 hydrogen bonds are found in the crystal structure. Crystal packing is stabilized mainly by van der Waals interactions (Fig. 2).

### Experimental

2-hydroxy-3-methoxybenzaldehyde (10 mmol) was added to a ethanolic solution of thiazol-2-amine (10 mmol). Then, the reaction mixture was heated for 2 h under reflux. After filtration a yellow powder was obtained. Suitable crystals for X-ray diffraction were obtained by recrystallization from methanol. Anal. Calcd (%) for C_11_H_10_N_2_O_2_S_1_ (Mr = 234.27): C,56.40; H, 4.30; N, 11.96; O, 13.66; S,13.69. Found (%): C,56.38; H, 4.35; N, 11.90; O, 13.69; S,13.68.

### Refinement

All the nonhydroxy H atoms were placed in geometrically idealized positions (C_methyl_—H = 0.96 and all other C—H = 0.93 Å) and constrained to ride on their parent atoms, with with *U*_iso_(H) = 1.2*U*_eq_(C) and 1.5*U*_eq_(C8). The H atoms attached to oxygen atoms were located from the Fourier differece map and refined isotropically.

### Crystal data

**Table:**

C_11_H_10_N_2_O_2_S	*F*(000) = 976
*M**_r_* = 234.27	*D*_x_ = 1.416 Mg m^−^^3^
Monoclinic, *C*2/*c*	Mo *K*α radiation, λ = 0.71073 Å
Hall symbol: -C 2yc	Cell parameters from 1208 reflections
*a* = 24.765 (3) Å	θ = 3.3–21.9°
*b* = 4.9619 (8) Å	µ = 0.28 mm^−^^1^
*c* = 20.238 (2) Å	*T* = 298 K
β = 117.931 (2)°	Block, colorless
*V* = 2197.2 (5) Å^3^	0.29 × 0.18 × 0.17 mm
*Z* = 8	

### Data collection

**Table:**

Siemens SMART CCD area-detector diffractometer	1920 independent reflections
Radiation source: fine-focus sealed tube	1139 reflections with *I* > 2σ(*I*)
graphite	*R*_int_ = 0.034
φ and ω scans	θ_max_ = 25.0°, θ_min_ = 1.9°
Absorption correction: multi-scan (*SADABS*; Sheldrick, 1996)	*h* = −29→26
*T*_min_ = 0.923, *T*_max_ = 0.954	*k* = −5→5
5338 measured reflections	*l* = −24→22

### Refinement

**Table:**

Refinement on *F*^2^	Primary atom site location: structure-invariant direct methods
Least-squares matrix: full	Secondary atom site location: difference Fourier map
*R*[*F*^2^ > 2σ(*F*^2^)] = 0.044	Hydrogen site location: inferred from neighbouring sites
*wR*(*F*^2^) = 0.110	H-atom parameters constrained
*S* = 1.02	*w* = 1/[σ^2^(*F*_o_^2^) + (0.0373*P*)^2^ + 1.8745*P*] where *P* = (*F*_o_^2^ + 2*F*_c_^2^)/3
1920 reflections	(Δ/σ)_max_ < 0.001
146 parameters	Δρ_max_ = 0.19 e Å^−^^3^
0 restraints	Δρ_min_ = −0.23 e Å^−^^3^

### Fractional atomic coordinates and isotropic or equivalent isotropic displacement parameters (Å^2^)

**Table:**

	*x*	*y*	*z*	*U*_iso_*/*U*_eq_	
N1	0.10966 (12)	1.1575 (6)	0.46446 (14)	0.0681 (8)	
N2	0.09882 (10)	0.9199 (5)	0.35514 (12)	0.0495 (6)	
O1	0.09126 (9)	0.6986 (4)	0.23318 (10)	0.0582 (6)	
H1	0.0833	0.8091	0.2575	0.087*	
O2	0.13089 (10)	0.3468 (4)	0.17130 (12)	0.0687 (6)	
S1	0.01303 (4)	1.2680 (2)	0.34433 (5)	0.0702 (3)	
C1	0.14555 (13)	0.7692 (6)	0.39241 (16)	0.0512 (7)	
H1A	0.1658	0.7842	0.4442	0.061*	
C2	0.16805 (12)	0.5776 (6)	0.35747 (15)	0.0458 (7)	
C3	0.13974 (12)	0.5484 (6)	0.27981 (16)	0.0457 (7)	
C4	0.16169 (13)	0.3571 (6)	0.24731 (17)	0.0514 (8)	
C5	0.21123 (14)	0.2002 (6)	0.29263 (19)	0.0608 (9)	
H5	0.2259	0.0724	0.2714	0.073*	
C6	0.23948 (14)	0.2313 (7)	0.3698 (2)	0.0642 (9)	
H6	0.2730	0.1246	0.3999	0.077*	
C7	0.21848 (13)	0.4170 (6)	0.40180 (18)	0.0576 (8)	
H7	0.2378	0.4370	0.4535	0.069*	
C8	0.14472 (19)	0.1291 (7)	0.1360 (2)	0.0966 (13)	
H8A	0.1856	0.1494	0.1428	0.145*	
H8B	0.1164	0.1291	0.0835	0.145*	
H8C	0.1415	−0.0380	0.1578	0.145*	
C9	0.08023 (13)	1.1000 (6)	0.39362 (16)	0.0500 (7)	
C10	0.07640 (17)	1.3433 (7)	0.48042 (19)	0.0747 (10)	
H10	0.0900	1.4100	0.5286	0.090*	
C11	0.02367 (16)	1.4240 (7)	0.42374 (18)	0.0678 (9)	
H11	−0.0031	1.5471	0.4274	0.081*	

### Atomic displacement parameters (Å^2^)

**Table:**

	*U*^11^	*U*^22^	*U*^33^	*U*^12^	*U*^13^	*U*^23^
N1	0.0747 (18)	0.082 (2)	0.0432 (15)	0.0066 (16)	0.0239 (14)	−0.0087 (14)
N2	0.0487 (15)	0.0539 (15)	0.0471 (14)	−0.0046 (13)	0.0234 (12)	−0.0034 (13)
O1	0.0626 (13)	0.0576 (13)	0.0500 (12)	0.0124 (11)	0.0227 (10)	−0.0032 (10)
O2	0.0912 (17)	0.0617 (15)	0.0625 (14)	0.0129 (12)	0.0438 (13)	−0.0047 (12)
S1	0.0629 (5)	0.0897 (7)	0.0526 (5)	0.0103 (5)	0.0227 (4)	−0.0114 (5)
C1	0.0543 (18)	0.0540 (19)	0.0412 (16)	−0.0111 (16)	0.0188 (15)	−0.0017 (15)
C2	0.0451 (17)	0.0415 (17)	0.0498 (18)	−0.0068 (14)	0.0214 (14)	0.0009 (14)
C3	0.0438 (17)	0.0396 (17)	0.0557 (19)	−0.0002 (14)	0.0250 (15)	0.0022 (15)
C4	0.0572 (19)	0.0481 (19)	0.058 (2)	−0.0037 (16)	0.0343 (17)	0.0010 (16)
C5	0.061 (2)	0.049 (2)	0.088 (3)	0.0018 (16)	0.048 (2)	0.0035 (18)
C6	0.0493 (19)	0.059 (2)	0.079 (3)	0.0027 (17)	0.0254 (18)	0.0138 (19)
C7	0.0516 (19)	0.056 (2)	0.0569 (19)	−0.0035 (16)	0.0189 (16)	0.0046 (17)
C8	0.162 (4)	0.068 (3)	0.084 (3)	0.021 (3)	0.077 (3)	−0.002 (2)
C9	0.0572 (18)	0.0507 (18)	0.0481 (18)	−0.0063 (15)	0.0298 (15)	−0.0029 (15)
C10	0.093 (3)	0.083 (3)	0.051 (2)	0.000 (2)	0.036 (2)	−0.018 (2)
C11	0.075 (2)	0.078 (2)	0.060 (2)	0.004 (2)	0.0391 (19)	−0.0082 (19)

### Geometric parameters (Å, °)

**Table:**

N1—C9	1.300 (3)	C3—C4	1.401 (4)
N1—C10	1.372 (4)	C4—C5	1.378 (4)
N2—C1	1.284 (3)	C5—C6	1.389 (4)
N2—C9	1.398 (3)	C5—H5	0.9300
O1—C3	1.351 (3)	C6—C7	1.361 (4)
O1—H1	0.8200	C6—H6	0.9300
O2—C4	1.361 (3)	C7—H7	0.9300
O2—C8	1.423 (4)	C8—H8A	0.9600
S1—C11	1.689 (3)	C8—H8B	0.9600
S1—C9	1.704 (3)	C8—H8C	0.9600
C1—C2	1.443 (4)	C10—C11	1.333 (4)
C1—H1A	0.9300	C10—H10	0.9300
C2—C3	1.397 (4)	C11—H11	0.9300
C2—C7	1.397 (4)		
			
C9—N1—C10	108.6 (3)	C7—C6—H6	119.8
C1—N2—C9	119.1 (2)	C5—C6—H6	119.8
C3—O1—H1	109.5	C6—C7—C2	120.5 (3)
C4—O2—C8	117.2 (3)	C6—C7—H7	119.8
C11—S1—C9	89.58 (16)	C2—C7—H7	119.8
N2—C1—C2	123.0 (3)	O2—C8—H8A	109.5
N2—C1—H1A	118.5	O2—C8—H8B	109.5
C2—C1—H1A	118.5	H8A—C8—H8B	109.5
C3—C2—C7	119.3 (3)	O2—C8—H8C	109.5
C3—C2—C1	121.0 (3)	H8A—C8—H8C	109.5
C7—C2—C1	119.7 (3)	H8B—C8—H8C	109.5
O1—C3—C2	122.8 (3)	N1—C9—N2	126.4 (3)
O1—C3—C4	117.3 (3)	N1—C9—S1	115.3 (2)
C2—C3—C4	119.9 (3)	N2—C9—S1	118.2 (2)
O2—C4—C5	125.7 (3)	C11—C10—N1	116.9 (3)
O2—C4—C3	114.9 (3)	C11—C10—H10	121.5
C5—C4—C3	119.4 (3)	N1—C10—H10	121.5
C4—C5—C6	120.5 (3)	C10—C11—S1	109.5 (3)
C4—C5—H5	119.7	C10—C11—H11	125.2
C6—C5—H5	119.7	S1—C11—H11	125.2
C7—C6—C5	120.4 (3)		

### Hydrogen-bond geometry (Å, °)

**Table:**

*D*—H···*A*	*D*—H	H···*A*	*D*···*A*	*D*—H···*A*
O1—H1···N2	0.82	1.91	2.627 (3)	146
